# Medical Education

**Published:** 1855-09

**Authors:** John Loche Chandler

**Affiliations:** St. Albans, Vermont


### ART. IV. — *Medical Education.* By John Loche Chandler, M. D., St. Albans, Vermont.

"Non ignara mali, miseris succurrere disco."

The plain English of my motto may be thus rendered — My early training in classical and scientific studies was almost nothing. Of the dead languages I am profoundly ignorant, with the exception of Latin, of which I know very little. Of living languages I am equally ignorant, with the exception of my mother-tongue; and even for the little skill I may claim in this, I find myself largely indebted to my superficial knowledge of Latin, a language I cannot read understandingly, even with the help of a dictionary. Of mathematics and philosophy, with all their dependencies, and natural history, I was utterly ignorant, when I most needed them as indispensable aids to the study of my profession. My acquirements in all these are yet so meagre, that in considering to what departments I may be most indebted for the little I have attained, I find myself too ignorant to determine. I simply know that numbers, magnitude, and their relations, are to be investigated and understood by the application of certain rules, or tests; but what these are exactly, or what they should be called, I know not. With the exception of some familiarity with a few good English Essayists, and Dramatists, and a little acquaintance with an ancient volume of Ethics, written by savans, of whom Isaiah, David, and Luke, a physician, are examples, such was my own preparation for entering on the study of medicine. "Fools rush in, where angels fear to tread."

So much for my "non ignarus mali;" and the plainness with which I have most truthfully stated it I trust will be tolerated, for the sake of enforcing more effectually on *such younger members of the profession*, as may need it, and who are not yet past hope, no less than are *medical students*, a just estimate of the importance of *preliminary* facilities, for acquiring a knowledge of medicine. Being myself well qualified to *feel* their indispensable necessity, "miseris succurrere disco;" which probably means, not liking my companionship, I would gladly rid myself of my fellow occupants, by helping them out of the ditch, upon my own shoulder. If *scholarly* readers choose to call my rendering of the passage a *free translation*, they can retire. I did not call their attention. *We* understand each other; that is, the "late-caught rustics" and myself, unscourged of birch, and unredolent of midnight oil, who are to consider these my lucubrations. Before they retire, however, let us do ourselves the justice, we, "the late-caught," to apprise them of our mindfulness, that their parchment-vouchers are but circumstantial evidence; that unconned lessons are sometimes *recited*, and that unused brains come to be little better than no brains.

Having the field exclusively to ourselves, let me warn you, confidentially, against yielding any vantage-ground, (we have little enough,) by presuming too much on the indolence of the enemy. The parchment-men have resources at their command that enable them, with comparative ease, to achieve that which costs us protracted, and sometimes fruitless toil; and though we are often tempted to undervalue the prowess of an educated competitor, from the fact of his palpable indolence and inattention to medical studies, emergencies frequently occur, which confound and put us to shame, by the demonstration of his superior tact in diagnosis; his deeper and more just conceptious of pathology; and his more enlightened views of therapeutic agencies. Whence comes all this? We have, perhaps, long observed his indolent habits, and have seldom, if ever, witnessed his exhibition of intellectual activity or force; while we are conscious ourselves of diligence and effort in professional inquiries. It unquestionably comes of early training in the preliminary studies, which are essential aids to the effective study of medicine; no less, perhaps, than of systematic training in the principles of medicine itself. With equal natural endowments, with habits of application, comparatively indolent, our competitor will long eclipse us, in the estimation of intelligent observers; so long, indeed, that most of us will tire of emulating the perseverance of the tortoise, in his fabled race with the hare. Such a race is, indeed, a hard one, and victory even, seems an inadequate reward for a life of hardship and self-denial. But the victory is sure, nevertheless; sure to those who will run diligently, and for whose benefit I am thus exposing my own deficiencies, and, perhaps, making myself ridiculous; not sure to those who, like myself, defer their efforts to repair the evils of a deficient education till they are fifty years old; and hopeless, if even after this late repentance, they suffer, like myself, long intervals of sottish indolence to intervene. With such, docility has not yet become an incompatibility; age has not yet benumbed their faculties with its petrifactions; they are yet in the early years of professional life; or better still, within the precincts of medical pupilage. To all such, having fair capacities, and who will work heartily in their calling, the future promises all that is honorable or desirable.

Two examples occur to me which I cannot deny myself the pleasure of exhibiting, as forcible illustrations of the power of indomitable purpose to overcome obstacles, apparently insuperable, in the path to usefulness and just professional distinction. They are both furnished in the history of two medical students, pupils of my father, the late Dr. Benjamin Chandler; who was quick to discover and appreciate talent, and watched its developments with the gusto of an epicure. The first of these presented himself to my father, an entire stranger, in the rough garb of a back-woods-man; announcing his wish to commence the study of medicine forthwith. It was the custom of country physicians, in those early days, to receive pupils, boarding, and sometimes even clothing them, trusting to their future professional success for remuneration. He signified his wish to discharge his pecuniary obligations, as they accrued, by his daily labor on the farm, or in any other employment my father might furnish. He had traveled some thirty miles on foot, from a new settlement among the mountains, where he was reared, and where his ardor in the pursuit of knowledge must have been kindled. Yet his training had been all effected in the rough and brief terms of the district schools of that pioneer period, and mountainous region, usually taught and sustained by back-woods men themselves. His bearing was indicative of intelligence and good sense; of solidity, rather than of brilliancy. My father acceded to his terms at once. It was during my own pupilage; and though the only advantage I could claim over him was a superficial smattering of Latin, I affected profound amazement at his temerity, in presuming to enter on the study of medicine with so little preparation; especially with the drawbacks on his time, by the undignified employment of "his own hands," in catering for his daily support. My father replied to this sage announcement of my sentiments toward my fellow pupil, that I should soon be relieved from the burden of such regrets, by finding myself amply employed in following, at a respectful distance, my fellow student's lead in the acquisition of knowledge. My impartial and sagacious father's prediction was ruefully verified. With no special claims to genius, he had intellectual strength, and an iron will, to do what he purposed; the true secret, no doubt, of success in every department of human pursuit. His work, was to diligently study and understand the elementary books in medicine, prescribed by his preceptor; his pastime, the entire fulfillment of his contract with my father; leaving him still many fragments of time, which were successfully appropriated in gleaning items of knowledge, from every department which could either directly, or indirectly facilitate the study of his profession. He is now an eminent, if not the most distinguished physician in a distant state; has been, I think, chairman on an important committee in our National Medical Association; at least, wrote the report of that committee, which was published in the Transactions of the association, and has given a volume to the profession which will be read and valued, long after his old chum is forgotten.

The late Dr. William Beaumont, of St. Louis, long distinguished as a surgeon in the army of the United States, and still more for his remarkable experiments and researches on the digestive function, was also the pupil of my father; of whom he was justly proud, and confidently predicted his future eminence in the profession. Dr. Beaumont also entered on the study of medicine under many disadvantages. He commenced somewhat later in life than is usual, and in addition to considerable deficiency in preliminary acquirements, labored under so great a degree of *deafness*, that serious apprehensions were entertained of its proving an insuperable obstacle to professional success. His own just estimate of these disadvantages may have given force and permanence to his efforts, working out, possibly, a higher grade of professional character, than would otherwise have been attained. The first years of his professional life were also consecrated, with rigid fidelity, to acquisition in such departments of science as he most needed; resulting in his early elevation to high rank in his profession, and ultimately leaving hundreds of his diplomated competitors immeasurably behind him.

For the deeper discoveries and the graver contributions to medical science, the profession must ever be mainly indebted to those who are fitted by *early* discipline, for vigorous and accurate thought. The analogies between physical and intellectual training are not mere mat ers of fancy, but have their foundation in truth. The country Jonathan, from very childhood wedded to his donkey, his plough, and his hoe; who covets no better pastime than a splinter of pine with his jack-knife, is not to be lightly esteemed. His individual service to his fellow men, who are nourished by the bread he produces, entitles him to no unenviable rank, as a public benefacter. But Jonathan may unwisely tire of his honorable employment, and choose to see the world. Some doggerel about "the wide, wide sea" may have caught his ear, and awakened a long dormant propensity; and he makes the sage discovery that he has "mistaken his mission;" that he has wasted long years in furrowing his father's acres, when he should have been ploughing "the mighty deep." His stripling brother, whose intellectual and physical capacities merely equal his own at the same age, catches the infection; and leaving the paternal hearth, and its genial pursuits, they enter together on the training which is to fit them for the duties, the endurance, and the dangers of a sailor's life. The stripling begins with the cabin-boy's berth, and Jonathan goes before the mast.

Their first essays in seamanship may be quite satisfactory. The lusty "yeo-heave" is, perhaps, not ill adapted to Jonathan's sturdy arm, well braced and consolidated by many a jerk and tug at the plough-tail. The flexile muscle and youthful alacrity of the stripling fit him well for the labyrinth of the cabin-boy's duty. But ere long, the hand of Jonathan, moulded and hardened to the plough tail, finds itself undocile in handling "the ropes." There are dizzy heights among the "masts," which are not only to be gained, but maintained. But "the *top* of his ambition" wabbles suspiciously. The unpliant muscle, staid and stark with its adaptations to the plough-tail and the rugged furrow, takes its lessons in agility hardly; the feet refuse to cling, and the fingers to twine, in their unwonted work. Jonathan looks upon the matter, however, as a sort of legerdemain, to be acquired by some mysterious method, which, no doubt, he shall master in due time, and he returns to his "yeo-heave" with redoubled energy; till his shipmates admit that his "yeo-heave" is unsurpassed, and he regards it himself, as the sum of seamanship. His comrades wink knowingly; or jeer, as he bestrides his hobby, whether in tempest or calm, nearing breakers, or in a clear sea; yet the incontrovertible fact that a good "yeo-heave" is an indispensable item in navigation, has secured his position as a sailor; and though he sports his tarpaulin unjauntily, he wears it with impunity.

Meantime the stripling's pliant fingers have become familiar with "the ropes;" his agile limbs and clear eye have been put in requisition, till he is at home and at ease, at every point in his "good ship," from mast-head to hold. His "yeo-heave" may be inferior to Jonathan's; but he wears his tarpaulin with unconscious jauntiness. On the next encounter of tempest and breakers, it would be no matter of surprise if the tug of Jonathan's sturdy arm, reinforced even with the "yeo-heave," should have less influence on the ship's course, than the agility, adroitness, precision and power, which is sure to characterize the stripling's execution of orders, in all emergencies.

> "The crampy shackles of the plough-boy's walk
> Tie the small muscles, when he strives to talk."

If disuse, or inappropriate adaptation in early life, disqualifies the tongue and its associate organs for the euphonies of an easy "enunciation," will "the late-caught rustic" and his intellectual faculties be any less encumbered with "shackles?" Will not these, also, have become too obdurate and intractable, by disuse, or perversion, for the purposes of effective, accurate thought; pliancy being no less important to its objects, than strength?

Let us follow Jonathan and the stripling in another direction. Instead of the doggerel about the "sea," some prating Siren may have gained their ears with high-flown jargon about "the godlike apprehension of man! how noble in reason! now infinite in faculties!" Man's physical dignity and importance is dwarfed at a blow; hand-craft is banished in disgrace, and head-craft becomes the cynosure. Alas! for Jonathan. ("Et quorum pars magna *fui*." *Sub rosa*.) His flagging and baffled intellect, galled with its yoke, and wincing with the pressure, may covet, when too late, honorable release from its responsibilities; and yearn for the jilted plough-tail, where it reposed in happy contemplation of the well-turned furrow, and its grateful returns. The stripling has also tasted the Siren's heady cup, and the learned professions constitute the domain, where the future obelisk is already rising, in the dim distance, for the inscription of their own names and deeds! But in what department shall it tower? The stripling dreams of the Bar, the Bench, and the Senate, where he is to distance all competitors, and captivate every auditor! Jonathan leans to the sagacious, and the profound; and already, in anticipation, nods his gracious acknowledgment to his deferential admirers. And what should shadow forth the grand ideal of his aspirations, if not the mysteries of Physic? The stripling's eloquence succumbs to Jonathan's wisdom, and they seek the field of medicine, but by different routes; the one, apparently direct—the other, circuitous.

Jonathan, though not *in the sere*, is "bearded like the pard," and consequently must spurn the milk by which unfledged striplings are nourished into manhood. He enters at once upon the intricacies of organization and functions; of general, descriptive, and pathological anatomy; but the very words in which structure, form, relation, function, and derangement are described, or defined, have an import which his own knowledge of language is inadequate to comprehend; and which no lexical expedient, within his reach, can help him to make clear. The originals of the figures, and examples, and processes, by which these are illustrated, belong to matters wholly beyond the range of his former inquiries; and even in mere descriptive anatomy, the definite force and clearness of the text is half lost to him. Jonathan marvels! But he ponders till he finds himself greatly comforted with the discovery that *the books* are the mere vestibule, the labyrinth of dark and winding passages, through which he is to grope for the *abracadabra*, the cabalistic key, which unlocks the great temple of medical science at once, and reveals all its mysteries, in full blaze. He resumes his courage, and encounters therapeutics. But here he is somewhat disappointed. Nature has comparatively so much, and art so little to do, in the prevention and cure of disease, that he almost takes it as a personal affront, and infringement on his own expected prerogative; and he regards Mistress Medicatrix, rather as a rival than a coadjutor. He inwardly thanks Heaven, however, that medicine is not wholly transcendental; that potions, pills, and powders, are veritable things, that can be tasted, touched, and handled; and he will triumphantly demonstrate that they can be swallowed.

Perhaps he may aspire to the honor of graduation in some metropolitan school of medicine? We might doubt his success; but the diploma will be forthcoming; for though his recitations may halt, and his thesis be a proxy, his *purse* is replenished from his father's acres. It will long remain a problem, whether his sojourn at the metropolis is for the better or the worse. The mind of the merest dullard doubtless, must feel the touch of intelligence from an attendance for months, on the instructions and demonstrations of the lecture-room, the dispensary, and the hospital. The principles of medicine, however, can no more be rightly taught, or apprehended, without certain prerequisites, than the philosophy of language and the graces of style can be taught or apprehended, without the previous knowledge of letters and words. His great deficiency in these preliminaries must of necessity result in his acquisition of superficial details, rather than principles. His pretension will outstrip even, his attainment in these, till it merges in temerity, and thus becomes all the more dangerous. Principles are the *ballast* of our imperfect *craft;* but temerity will stretch its canvas to the gale, inversely with its amount of deposit in the *hold.*

Jonathan is diplomated, and takes his place in the profession, where, to do him justice, he sometimes deserves his success, in supplanting a competitor of still more exceptionable claims. But what are his characteristics as a physician? Instead of delving the mine for ore, he traverses the surface, and is ever adding to his hoard of gathered trifles, the shapeless bricks and curious implements, which sound experience had already tested, and cast away as worthless. In the exercise of his professional function, instead of considering attentively the known laws of the animal economy, and quietly seeking for the evidence and nature of their disturbance, he hurries at once, from expedient to expedient; peers furtively, hither and thither, for *remedies*, all unconscious the while, that the *ailment* to which he ministers is yet a *terra*

*incognita*, in his own mind; and blind to the palpable absurdity of fabricating means, without the knowledge of ends. Hobbies in medicine, unfortunately, are more abundant, and more mischievous, than in navigation. If Jonathan the sailor bestrided his hobby on every emergency at sea, it will be no marvel if Jonathan the Doctor, should mount his "yeo-heave," for a steeple chase in medicine. It is no common continence that can refrain, when conscious impotence quails before pressing emergency. The windy jade, invitingly caparisoned, the very stirrup swaying as if by magic, to the fitting foot, may, under like circumstances, prove a sore temptation to us. It ill becomes us to scoff, while Jonathan rides. No doubt, he will improve. Intercourse with intelligent men, and familiarity with the practice of others will gain him the endurance of the profession, and the tolerance of his patrons. But "the crampy shackles" will "tie" him still, and mark the type of his whole professional life.

But what of the stripling? Happily, his chin was yet innocent of down, and consequently there were no incompatibilities between himself and the birchings which appertain to the school room, the seminary, or the college Birch is fragrant; and where it enforces diligence, it sheds a grateful and refreshing odor. The stripling took his discipline kindly, and with such singleness of heart, that he came to be unconscious, even, of the obelisk and its blazon. I am in no mood to follow him, in his progress onward, from the preparatory schools to the university; and thence, through the halls of scientific medicines, to his profession. How should I? My foot is unused to such acclivities. *Kith and kin* forbid, and prompt me to another path. "The soul of Jonathan was knit with the soul of David; and Jonathan loved him as his own soul." To me, the stripling is an alien, and the history of his pupilage may be written, and his professional characteristics portrayed, by some kindred Bigelow, or Holmes, if they choose, but not by me. I merely chronicle the fact, that Jonathan was no churl in his welcome of the younger brother to a common field of labor. He was patronizing and protective in his bearing toward him, as became an elder brother, though it often occurred that the touch of the stripling's finger achieved the purpose for Jonathan's patient, that had long resisted the sturdy "yeo heave."

The effective study of medicine involving the exercise of the mental powers, some just conceptions of the constitution, or economy of these powers, must be necessary to their right use. I may be wasting words upon others, but I am conscious that what I now conceive to have been erroneous opinions on this subject, were serious obstacles to my own progress in professional attainment. Many distinctions have been attempted to designate the difference between genius and talent, which I conceive are too vague to be of much practical value. My own perceptions are too gross to appreciate the transcendental chemistry by which the schoolmen have analyzed the human intellect, till each ultimate iota of its faculties is set apart; till the last barley-corn which found itself balancing doubtfully on *ultima thule* is subjugated, and falls discreetly into its place, with the prim precision of a nosological arrangement. With all deference to the Psychologists, in their attempts to anatomize the intellect; to dissect its several systems; to demonstrate each specific organ, they seem to have acted on the unwarranted assumption that the faculties of the mind, like the functions of the body, are each dependent on the agency of a specific intellectual organ; as the function of sight is dependent on the physical organization of the eye, or hearing on that of the ear. Physiologists have succeeded, in the main, in demonstrating the dependence of each particular function on its appropriate organ; and have been so distinctive in their definitions, that little danger remains of our mistaking the manifestations of one, for those of another. But it is noticeable that one faculty, or manifestation of mind is often defined by terms, or phrases, which are as often used, and with equal appropriateness, to designate another. I know not that the masters in psychology advocate such analogy between mental and physical organization, for 1 never read them. I simply know that the second-rate literature of the day, with which, I am sorry to say, I have been most conversant, and to which I fear young men are generally most addicted, abounds with loose phraseology, and crude opinions, leading to the conclusion that each separate act, or process of the intellect, is effected exclusively by some isolated or specific faculty of the mind, and not resulting from the action of the mental powers, *as a whole.* Analogies no doubt may be found, or fancied, between the most dissimilar things; but on the plea of analogy, we might as well ascribe the phenomena of vital action to the power of gravitation, it being the law which regulates the motions of the visible universe, as to claim the dependence of each intellectual faculty on a special mental organ, the several functions of the living body being dependent, each on its special organ. A better illustration would be, to liken the intellect to the *single string*, which the finger of Ole Bull has demonstrated to be adequate for unlimited variety and power of intonation.

The power of invention, which seems to have been claimed exclusively, as the definition of genius, from its very nature must include, and be identical with, the elements which constitute the power to improve, to adapt, to execute, being the definition claimed for talent, would therefore seem to favor the conclusion that genius and talent are mere properties, moods, or manifestations of one intellectual principle. Cultivation of the mind, no doubt, is more effectual in the development of what is called talent, than of genius, though manifestly not inoperative on the latter. Indeed, I see not how it is possible to *cultivate* genius, except by the methods which all admit are indispensable to the development of talent; and while we are idly admiring and coveting the power of the former, we are wasting our resources, and insuring our own defeat, by spurning the humble achievements, which constitute the step-stone to the higher glory. The strength of talent is of more intrinsic worth than the brilliancy of genius.

I do not propose to offer a programme of subjects, nor of medical books, for the guidance of medical students, or the younger members of the profession. It is the province of educated men. My purpose is simply to suggest those aids and expedients, which separately may seem trifles, but in the aggregate prove invaluable, and perhaps, indispensable, to the full appreciation of the books and medicine. *Language* is the vehicle, and to some extent even, an instrument of thought. It might be too much to claim that a good thinker, of necessity presupposes an adept in language; but we may safely assume that, *other things being equal*, the accuracy and force of our thought will be in proportion to our knowledge and skill in language. Single words have frequently such diversity of meaning, and phrases such variety of import, depending on their relations with other words and phrases; and often, indeed, varied by the subject on which they are employed, that two minds, of equal capacity, and giving equal heed, may draw very different conclusions from the same lecture, or the same chapter, and yet both be wrong. A very little knowledge of Latin, my own experience has proved, greatly facilitates our apprehension of the force and import of English words. If *so much* is not already attained, it should be attempted. A little success will amply repay all the sacrifice it might require. A large amount of professional terms in medicine are derived from the Greek; and these often compounded from several roots, of diverse import. Being wholly ignorant of Greek, myself, as a consequence, I have probably expended more extra time, during my life, in turning the leaves of all sorts of Lexicons, to attain the required elucidation, than goes to the *legal* term of medical pupilage; and what was still more disheartening, often unsuccessfully. Though I am far from regarding our ignorance of Greek and Latin, as an insurmountable bar to fair attainments in our profession, I have little doubt that six months, devoted to the study of these languages, under a competent teacher, during pupilage, or even in the early years of practice, would be repaid by four-fold facilities in learning aright the principles of medicine. The benefit is not all comprised in the disclosure of the literal import of terms, compounded from Greek and Latin roots; but in the subjugation of the mind to the power and significance of cultivated language, thus pervading and irradiating the whole range of study. Indeed, if there be one indispensable prerequisite for the successful study of medicine, it is knowledge and skill in the *language* in which it is presented to the mind of the student; the master-key to all the learning which the same language furnishes for its elucidation.

If the young practitioner, whose early training has been stinted, expects to repair the evil, he must secure facilities, both in regard to time and place, for study. He must also enforce upon himself a *habit* of study, which, unfortunately his training never can have fully formed. A habit of study in its appropriate time and place, will beget a habit of observation and reflection, without which, progress in professional knowledge and skill is impossible. When the appropriated hour arrives, professional engagements permitting, he should be found in his place, and at his table; which should be furnished, as its permanent and indispensable appendages, with the best dictionaries, "unabridged," and without stint; English, classical, medical, surgical; and the more the better, in each department. Compends in medicine are dangerous toys, but compends in other departments of science, needed for reference, and often indispensable for elucidating passages in medical books, are, perhaps, admissible. The medical periodicals should never be overlooked; indeed cannot be wholly disregarded by the medical practitioner, young or old, without loss, and even retrogradation, in his professional position. It might even be a judicious improvement in medical education, to add the exaction of some familarity with these, to the pupil's programme.

It should be borne in mind, however, that the intellect can never be disciplined to practical efficiency in medicine, by exclusive professional habitude. The mental powers, wearied with protracted effort in one direction, are not merely rested, but refreshed with new accessions of vigor, by occasional employment in some other direction. The hyper-fecundity of the age in works of fiction, farce, and foolery, makes the proper selection of books a most difficult achievement. Nineteen-twentieths of the current literature of the day would be more fitly appropriated as fuel for the fire, than as food for the mind. Fortunately for us, right-minded and intelligent benevolence, coöperating with enterprise in trade, has provided the means of furnishing our table without danger of loading it with obtrusive frippery, mawkish sentiment, or moral pollution. The "Living Age," offers an example of sound eclecticism in literature, judiciously conservative, and worthy of all imitation. The young physician will soon have attained no small amount of the general knowledge, and have done something in the effective mental culture, which, his professional necessities require, by the careful perusal of its weekly numbers.

The exercise of reducing our own thoughts to *written* language, will be found a useful method of cultivation. But it should be borne in mind that thoughts, to be written, must preëxist in the mind. It does not follow, however, that an attempt to write should always be deferred till we are conscious of the thought; for the effort itself may be the very stimulus required, to rouse the torpid intellect to activity. It may be well to consider the question, meanwhile, somewhat deliberately, whether our manuscript shall be dispatched at once, to a Medical Journal! Dr. J. Bigelow's just rebuke should first be pondered: "Medical journals are filled with the crude productions of aspirants to the cure of diseases." The pen should be used to minister to our own cultivation; not to our self-conceit. The same writer has condensed a volume into a sentence, on the subject of medical education, before which my own pretension should be dumb. "The usefulness of a medical school depends not so much on the length of its session, as upon the amount of education, primary and ultimate, which it requires, the fidelity with which it exacts its own professed requisitions, and the train of healthy exertion, active inquiry, and rigid, methodical study, to which it introduces its pupils."

